# Notice to Delegates—Railway Transportation to the New York November Congress on Tuberculosis—One Fare and a Third Round Trip to New York and Return

**Published:** 1906-10

**Authors:** Clark Bell

**Affiliations:** Treas. and Sec. of Council, 39 Broadway, New York City


### NOTICE TO DELEGATES—RAILWAY TRANSPORTATION TO THE NEW YORK NOVEMBER CONGRESS ON TUBERCULOSIS—ONE FARE AND A THIRD ROUND TRIP TO NEW YORK AND RETURN.

---

The management announces that arrangements have been concluded with the Railway Trunk Line Association, by which members and delegates can secure transportation for the round trip on the certificate plan, at one fare and one-third to New York and return, for the American International Congress on Tuberculosis, November 14, 15, and 16, 1906, provided at least 100 persons shall have obtained the necessary certificate and paid the fare one way.

Special care must be taken to secure the certificate from the railway agent before starting. Without this certificate the return ticket can not be obtained.

Members and delegates wishing to avail themselves of reduced rates will please send their names and address to

CLARK BELL, ESQ.,
Treas. and Sec. of Council, 39 Broadway, New York City.

